# Interaction between gender and post resuscitation interventions on neurological outcome in an asphyxial rat model of cardiac arrest

**DOI:** 10.1186/s12872-021-02262-5

**Published:** 2021-09-16

**Authors:** Jianjie Wang, Jingru Li, Bihua Chen, Yiming Shen, Juan Wang, Kaifa Wang, Changlin Yin, Yongqin Li

**Affiliations:** 1 Department of Biomedical Engineering and Imaging Medicine, Army Medical University, Chongqing, 400038 China; 2 Department of Emergency, Chongqing Emergency Medical Center, Chongqing, 400014 China; 3 Department of Emergency, Southwest Hospital, Army Medical University, Chongqing, 400038 China; 4 Department of Intensive Care, Southwest Hospital, Army Medical University, Chongqing, 400038 China

**Keywords:** Cardiac arrest, Cardiopulmonary resuscitation, Hydrogen inhalation, Neurological outcome, Gender, Target temperature management

## Abstract

**Purpose:**

Previous clinical studies have suggested an effect of gender on outcome after out-of-hospital cardiac arrest, but the results are conflicting and there is no uniform agreement regarding gender differences in survival and prognosis. The present study was aimed to investigate the interaction between gender and post resuscitation interventions on neurological outcome in an asphyxial rat model of cardiac arrest.

**Methods:**

Asphyxia was induced by blocking the endotracheal tube in 120 adult Sprague–Dawley rats (60 males and 60 females) at the same age. Cardiopulmonary resuscitation (CPR) was started after 5 min of untreated cardiac arrest. Animals were randomized into one of the three post resuscitation care intervention groups (n = 40, 20 males) immediately after resuscitation: (1) normothermic control (NC): ventilated with 2% N_2_/98% O_2_ for 1 h under normothermia; (2) targeted temperature management (TTM): ventilated with 2% N_2_/98% O_2_ for 1 h under hypothermia; (3) hydrogen inhalation (HI): ventilated with 2% H_2_/98% O_2_ for 1 h under normothermia. Physiological variables were recorded during the 5 h post resuscitation monitoring period. Neurological deficit score (NDS) and accumulative survival were used to assess 96 h outcomes. Mutual independence analysis and Mantel–Haenszel stratified analysis were used to explore the associations among gender, intervention and survival.

**Results:**

The body weights of female rats were significantly lighter than males, but CPR characteristics did not differ between genders. Compared with male rats, females had significantly lower mean arterial pressure, longer onset time of the electroencephalogram (EEG) burst and time to normal EEG trace (TTNT) in the NC group; relatively longer TTNT in the TTM group; and substantially longer TTNT, lower NDSs, and higher survival in the HI group. Mutual independence analysis revealed that both gender and intervention were associated with neurological outcome. Mantel–Haenszel stratified analysis demonstrated that female rats had significantly higher survival rate than males when adjusted for the confounder intervention.

**Conclusion:**

In this rat model cardiac arrest and CPR, gender did not affect resuscitation but associated with neurological outcome. The superiority of female rats in neurological recovery was affected by post resuscitation interventions and female rats were more likely to benefit from hydrogen therapy.

**Supplementary Information:**

The online version contains supplementary material available at 10.1186/s12872-021-02262-5.

## Introduction

Out-of-hospital cardiac arrest (OHCA) remains a major public health issue and the most common cause of death all over the world. Approximately around 544,000 Chinese, 275,000 Europeans and 350,000 Americans suffer cardiac arrest every year with an overall survival rate less than 10% [1, 2]. Key factors associating with the return of spontaneous circulation (ROSC) are cause of arrest, initial shockable rhythm, provision of bystander cardiopulmonary resuscitation (CPR), ambulance response time, CPR duration and endotracheal intubation. Key factors associating with the prognosis of neurological outcomes are advanced life support and post resuscitation care [3]. The resuscitation efforts in response to these factors are therefore considered to be a major challenge for emergency medical services because of the high incidence of cardiac arrest. In order to optimize the clinical practice and improve outcomes, the latest American Heart Association (AHA) Guidelines for CPR and Emergency Cardiovascular Care suggested the chain of survival concept with fix links. These include activation of the emergency response, provision of high-quality CPR, early defibrillation, advanced life support, effective post resuscitation intervention, and recovery [4].

Recently, an increasing number of studies have focused on the effect of gender on outcome of cardiac arrest [5, 6]. The epidemiological investigation results showed that female and male victims differed significantly in characteristics of baseline and CPR. Compared with male victims, the female patients are tend to be older, arrest more in their home, present with more non-shockable rhythms, with more comorbidities, with fewer witnessed, have longer response time, less likely to receive bystander CPR and post resuscitation interventions [7]. With respect to hospital discharge and neurological outcome, however, conflicting results were reported and there is no uniform agreement regarding gender differences in survival and prognosis. Some of the studies showed that female victims had better outcomes [8], other studies demonstrated that female victims had similar outcomes [9], while the other studies reported that female victims had worse outcomes compared with males [10]. Unfortunately, the majority of these studies examining the association between gender and outcomes included population with different inclusion criteria or specific subsets. Therefore, it is still unclear whether these divergent findings are due to gender related female hormones or due to difference in characteristic of CPR and/or post resuscitation interventions.

The present study was designed to investigate the interaction between gender and post resuscitation intervention on neurological outcome in an asphyxial rat model of cardiac arrest and CPR. We hypothesized that neurological outcome is independent of gender. We further hypothesized that post resuscitation interventions have no effect on the association between gender and outcome.

## Methods

### Animal preparation

The experimental protocol was approved by the Laboratory Animal Welfare and Ethics Committee of the Army Medical University. One hundred twenty (60 males and 60 females) healthy adult Sprague–Dawley rats aging between 10 and 12 weeks supplied from laboratory animal center of the Army Medical University were used for this study. The study was in accordance with the ARRIVE guidelines and all animals received humane care in compliance with the Principles of Laboratory Animal Care and Guide for the Care and Use of Laboratory Animals.

All animals were housed under controlled laboratory conditions with free access to chow and water. Animals were fasted overnight and anesthetized with intraperitoneal injection of pentobarbital sodium (45 mg/kg). Additional doses (10 mg/kg) were administered to maintain anesthesia when the animals showed signs of recovery or wake up. Mechanically ventilation was supplied with a tidal volume of 0.65 ml/100 g at a FiO_2_ of 0.21 (ALC-V8, Alcott Biotech Co. Ltd, Shanghai, China) after endotracheal intubation. Three subcutaneous needle electrodes were inserted into the limbs for electrocardiogram (ECG) measurement. Four subdermal needles were inserted into the surfaces of the skull for electroencephalogram (EEG) measurement. Two PE-50 catheters were cannulated from the right femoral artery and left femoral vein for arterial blood pressure monitoring and drug delivery. Core temperature was monitored using an esophageal probe (TH-212, Bjhocy Science and Technology Co Ltd, Beijing, China) and maintained at 37.0 ± 0.5 °C with a lamp in preparation phase.

### Experimental procedures and randomization

The experimental procedures were established in our previous studies [11, 12]. Briefly, a single dose of pipecuronium bromide (0.1 mg/kg) was administered after baseline collection. Asphyxia was induced by disconnecting the mechanical ventilator and clamping the endotracheal tube. Cardiac arrest was defined as a mean arterial pressure (MAP) less than 20 mmHg and usually occurred approximately 3 min after asphyxia induction. CPR was started after 5 min of untreated cardiac arrest with a compression rate of 200/min, a depth of 25% diameter of the chest, a ventilation frequency of 80/min and a FiO_2_ of 0.98. Quality of CPR was controlled by maintaining the arterial diastolic blood pressure at more than 20 mmHg. A dose of epinephrine (0.02 mg/kg) was administered 30 s after the start of CPR. A single 2 J defibrillation (M-Series, Zoll Medical Corporation, Chelmsford, MA, USA) was delivered if the cardiac rhythm was shockable. Return of spontaneous circulation (ROSC) was defined as a supraventricular rhythm with MAP more than 60 mmHg and lasted for at least 5 min.

After resuscitation, animals were randomized into one of the three post resuscitation care intervention groups (n = 40, 20 males and 20 females) using the sealed envelope technique: (1) normothermic control (NC): ventilated with 2% N_2_/98% O_2_ for 1 h and then with room air under normothermia; (2) targeted temperature management (TTM): ventilated with 2% N_2_/98% O_2_ for 1 h and then with room air under TTM. TTM was composed of hypothermia (33 °C) induction (10 min), maintenance (2 h) and rewarming (2 h) phases; (3) hydrogen inhalation (HI): ventilated with 2% H_2_/98% O_2_ for 1 h and then with room air under normothermia. The investigator was blind to the experimental group during CA/CPR procedure but did not blind to post resuscitation care because he gave the intervention. After 5 h intensive care, all catheters and tubes were removed and wounds were surgically sutured. Animals were returned to their cages for 96 h neurological outcome assess. The animals were euthanized by a lethal intraperitoneal injection of sodium pentobarbital (150 mg/kg) at the end of the experiment.

### Measurements

Arterial pressure, ECG, and EEG waveforms were constantly measured and recorded for 5 h using a PC-based data acquisition system supported by WINDAQ software (DATAQ Instruments Inc., Akron, OH, USA). Left ventricular ejection fraction (EF), the indicator of myocardial contractility, was noninvasively assessed at baseline and at hourly intervals after resuscitation using an echocardiograph system (DC-6, Mindray Medical International Limited, Shenzhen, China).

The characteristics of the earlier post resuscitation EEG, including the onset time of the EEG burst (OTOB) and time to normal EEG trace (TTNT) were quantitatively analyzed [12]. OTOB was defined as the time from ROSC to the initial burst suppression pattern, and TTNT was defined as the time from ROSC to the initial continuous background pattern (Additional file 1: Fig. S1). The neurological deficit score (NDS) was examined 24, 48, 72 and 96 h after ROSC by two investigators blinded to the treatment using a NDS system (0–500 scale; 0 means no observed neurological deficit, 500 represents death or brain death) that was developed to evaluate neurological appearance after global cerebral ischemia for rats [13]. Animals were closely observed and the recovery condition was regularly evaluated by the investigators, every 2 h during day time and every 4 h during night time. If an animal was found to be dead at a specific observation time, the death time would be inferred as 1 (day) or 2 h (night) ago. The 96 h survival times were recorded for cumulative overall survival analysis.

### Statistical analysis

The normal distribution of the data was confirmed using the Kolmogorov–Smirnov test. Normal and abnormal distribution data were reported as mean ± standard deviation and median including interquartile range. Single physiological measures were analyzed using one way ANOVA. Repeated physiological measures were analyzed using the general linear models approach. NDSs were analyzed non-parametrically using Kruskal–Wallis test. The Kaplan–Meier analysis and the log-rank test were used for survival analysis. Mutual independence of gender, treatment and outcome was tested in a three-dimensional contingency table, as proposed by Everitt et al. [14]. The estimates of frequencies to be expected when the hypothesis is true were firstly calculated. The estimates of frequencies were then compared with the observed frequencies by means of the usual chi-square statistic. The chi-square statistic was finally compared with the tabulated critical value having the relevant number of degrees of freedom. If the chi-square statistic was greater than the critical value, the hypothesis of mutual independence would be rejected and partial independence would be further analyzed. Otherwise, there were no significant associations between all variables and further analysis of the table is unnecessary. Mutual independence of variables was tested by means of usual chi-square statistic and Mantel–Haenszel stratified analysis. A *p* < 0.05 was considered statistically significant.

## Results

There were no differences in baseline physiological measurements and resuscitation data among the three experimental groups. However, female rats had significantly lighter body weight, required less pentobarbital and epinephrine compared with that of males in each intervention group (Tables 1, 2 and 3). All animals were successfully resuscitated and no statistical differences in asphyxia time required to induce cardiac arrest, duration of CPR and number of defibrillation shocks between female and male rats.

**Table 1:** Baseline and cardiopulmonary resuscitation (CPR) characteristics between male and female rats in the normothermic control group

Measurement	Male (n = 20)	Female (n = 20)	*p* value
Body weight (g)	291.9 ± 24.9	236.9 ± 24.4	< 0.001
Heart rate (BPM)	430.5 ± 43.6	418.8 ± 27.8	0.317
MAP (mmHg)	131.1 ± 10.6	132.3 ± 8.3	0.704
Temperature (℃)	36.9 ± 0.1	36.9 ± 0.2	0.257
Asphyxia time (s)	175.2 ± 26.7	174.6 ± 31.3	0.948
CPR duration (s)	76.3 ± 17.4	82.6 ± 23.3	0.340
Defibrillations (n)	0.1 ± 0.2	0 ± 0	0.330
Pentobarbital (mg)	37.6 ± 5.7	28.2 ± 4.7	< 0.001
Epinephrine (µg)	5.8 ± 0.5	4.7 ± 0.5	< 0.001

*BPM* Beats per minute, *MAP* mean arterial pressure

**Table 2:** Baseline and cardiopulmonary resuscitation (CPR) characteristics between male and female rats in the targeted temperature management group

Measurement	Male (n = 20)	Female (n = 20)	*p* value
Body weight (g)	308.7 ± 26.6	245.7 ± 14.3	< 0.001
Heart rate (BPM)	413.9 ± 52.0	407.1 ± 41.1	0.649
MAP (mmHg)	127.4 ± 13.9	125.1 ± 15.7	0.635
Temperature (℃)	37.0 ± 0.2	37.0 ± 0.3	0.639
Asphyxia time (s)	188.5 ± 43.4	179.4 ± 32.9	0.457
CPR duration (s)	82.2 ± 16.7	75.7 ± 20.9	0.289
Defibrillations (n)	0.1 ± 0.3	0 ± 0	0.163
Pentobarbital (mg)	40.0 ± 9.2	29.7 ± 4.2	< 0.001
Epinephrine (µg)	6.2 ± 0.5	4.9 ± 0.3	< 0.001

*BPM* Beats per minute, *MAP* mean arterial pressure

**Table 3:** Baseline and cardiopulmonary resuscitation (CPR) characteristics between male and female rats in the hydrogen inhalation group

Measurement	Male (n = 20)	Female (n = 20)	*p* value
Body weight (g)	288.3 ± 24.0	236.5 ± 21.8	< 0.001
Heart rate (BPM)	433.8 ± 37.5	418.3 ± 48.8	0.267
MAP (mmHg)	133.2 ± 7.9	131.7 ± 8.4	0.551
Temperature (℃)	37.0 ± 0.1	36.9 ± 0.1	0.253
Asphyxia time (s)	170.9 ± 21.1	172.9 ± 30.1	0.809
CPR duration (s)	84.5 ± 16.2	78.8 ± 14.6	0.254
Defibrillations (n)	0.1 ± 0.3	0.2 ± 0.7	0.764
Pentobarbital (mg)	37.3 ± 11.6	26.4 ± 3.5	< 0.001
Epinephrine (µg)	5.8 ± 0.5	4.7 ± 0.4	< 0.001

*BPM* Beats per minute, *MAP* mean arterial pressure

### Effects of gender on outcome for NC

**Fig. 1:**
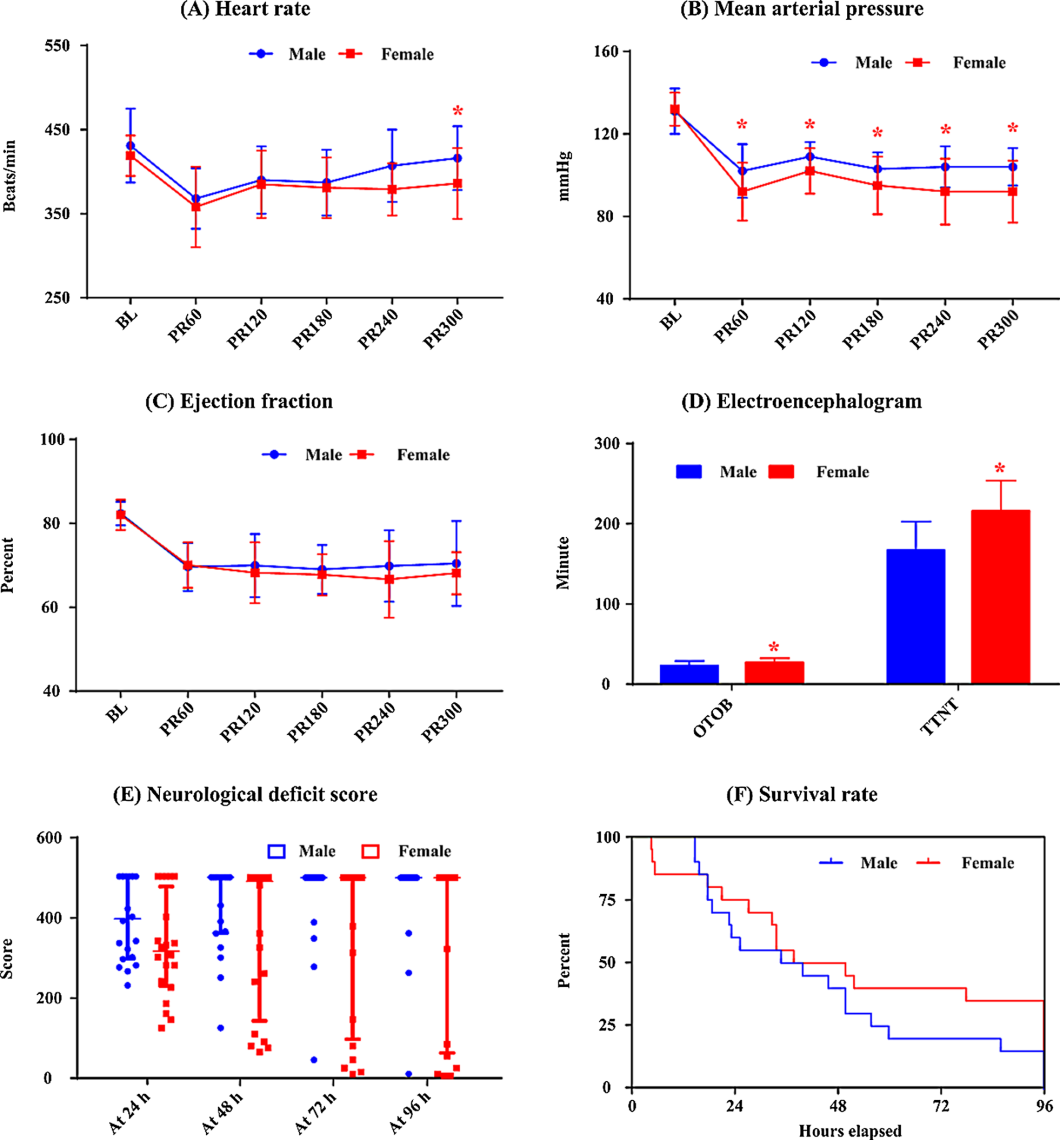
The comparison of heart rate (**a**), mean arterial pressure (**b**), left ventricular ejection fraction (**c**), electroencephalogram (**d**), neurological deficit score (**e**) and Kaplan–Meier survival curve (**f**) between female and male rats in the normothermic control group. BL: baseline; PR60, PR120, PR180, PR240 and PR300: 60, 120, 180, 240 and 300 min post resuscitation; OTOB: onset time of the electroencephalographic burst; TTNT: time to normal electroencephalographic trace; *: *p* < 0.05 compared with males

Figure 1 shows the hemodynamic and neurological data between female and male rats during the post resuscitation period for animals subjected to NC group. Compared with male rats, heart rate at 5 h post resuscitation (386.6 ± 41.9 vs. 415.7 ± 38.1 beats/min, *p* = 0.022) and MAP during the post resuscitation period (91.8 ± 14.2 vs. 102.4 ± 13.3 mmHg, *p* = 0.019; 101.7 ± 11.4 vs. 108.8 ± 7.4 mmHg, *p* = 0.025; 95.3 ± 13.7 vs. 103.3 ± 8.2 mmHg, *p* = 0.033; 91.7 ± 16.0 vs. 103.8 ± 10.0 mmHg, *p* = 0.007; 91.6 ± 14.9 vs. 103.6 ± 9.4 mmHg, *p* = 0.005) were significantly lower, while OTOB (26.9 ± 5.3 vs. 22.7 ± 6.2 min, *p* = 0.028) and TTNT (215.6 ± 38.1 vs. 166.6 ± 36.2 min, *p* < 0.001) were significantly higher in females rats. Female rats had a relative higher survival rate than male rats (35.0 vs. 15.0%, *p* = 0.144) but did not have statistical significance. Additionally, no differences in EF and NDS and were observed between female and male rats (Additional file 1: Table S1).

### Effects of gender on outcome for TTM

**Fig. 2:**
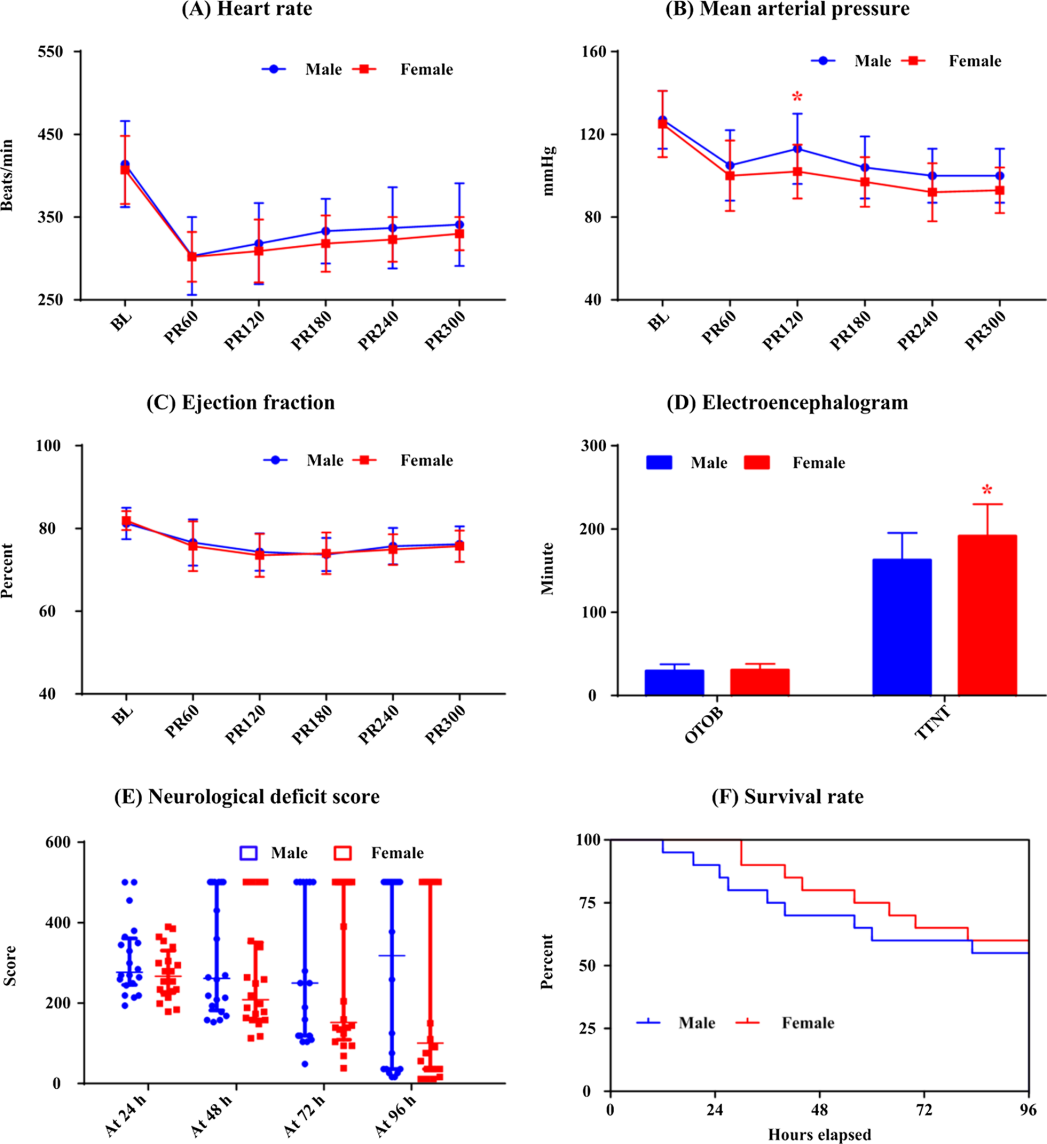
The comparison of heart rate (**a**), mean arterial pressure (**b**), left ventricular ejection fraction (**c**), electroencephalogram (**d**), neurological deficit score (**e**) and Kaplan–Meier survival curve (**f**) between female and male rats in the targeted temperature management group. BL: baseline; PR60, PR120, PR180, PR240 and PR300: 60, 120, 180, 240 and 300 min post resuscitation; OTOB: onset time of the electroencephalographic burst; TTNT: time to normal electroencephalographic trace; *: *p* < 0.05 compared with males

Figure 2 presents the post resuscitation measurements and neurological outcomes for animals subjected to TTM group. There were no differences in heart rate, MAP, EF, EEG, NDS and survival rate between female and male rats, except that MAP measured at 120 min after resuscitation was significantly lower (102.3 ± 12.5 vs. 112.6 ± 16.7 mmHg, *p* = 0.033) and TTNT (191.6 ± 38.2 vs. 162.6 ± 32.8 min, *p* = 0.014) was significantly longer in female rats.

### Effects of gender on outcome for HI

**Fig. 3:**
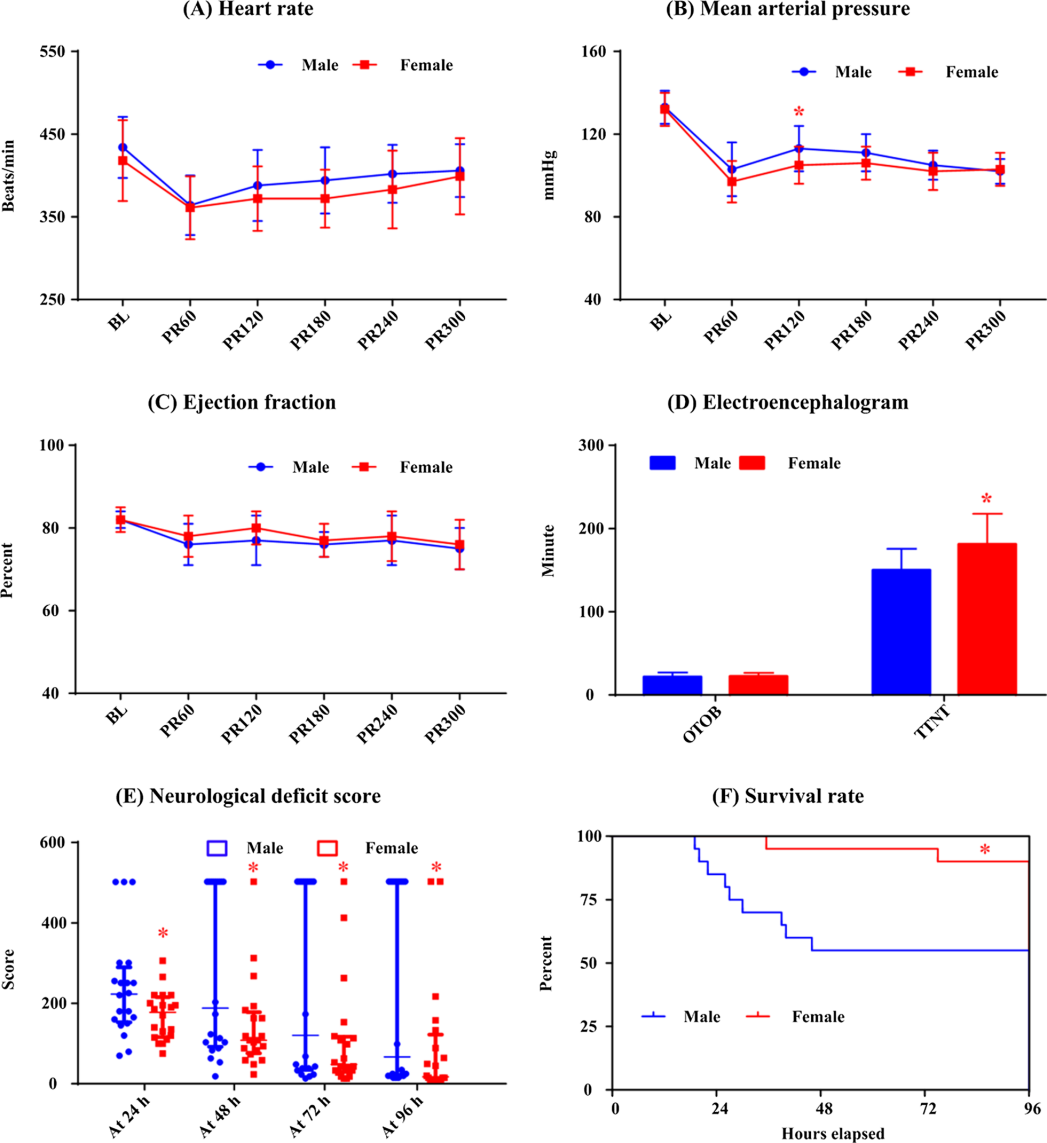
The comparison of heart rate (**a**), mean arterial pressure (**b**), left ventricular ejection fraction (**c**), electroencephalogram (**d**), neurological deficit score (**e**) and Kaplan–Meier survival curve (**f**) between female and male rats in the hydrogen inhalation group. BL: baseline; PR60, PR120, PR180, PR240 and PR300: 60, 120, 180, 240 and 300 min post resuscitation; OTOB: onset time of the electroencephalographic burst; TTNT: time to normal electroencephalographic trace; *: *p* < 0.05 compared with males

Figure 3 lists the hemodynamic data and neurological outcome for animals subjected to HI group. No differences in heart rate and EF were observed between female and male rats. However, MAP measured at 120 min after ROSC was significantly lower (104.9 ± 9.5 vs. 113.3 ± 10.5 mmHg, *p* = 0.012) and TTNT was significantly higher (181.8 ± 36.5 vs. 149.9 ± 25.5 min, *p* = 0.003) in female rats. Additionally, NDS was significantly lower during the 4 days following resuscitation and survival rate (90.0% vs. 55.0%, *p* = 0.013) was significantly higher in female rats compared with that of male rats (Additional file 1: Table S1).

### Interaction between gender and post resuscitation intervention on neurological outcome

Table 4 shows the three-dimensional contingency table to explore the interaction between gender and post resuscitation intervention on 96 h outcome. As shown in Table 5, mutual independence testing indicated that there were significant associations among gender, intervention and outcome. Partial independence testing demonstrated that outcome was not independent of gender and intervention, intervention was not independent of gender and outcome, whereas gender was independent of intervention and outcome. Since gender was independent of intervention and outcome, male and female groups were emerged when comparing the outcomes of different intervention group. A total of 10, 23 and 29 rats were survived in the NC, TTM and HI groups (n = 40 in each group, 20 males and 20 females). Animals treated with TTM (57.5% vs. 25.0%, *p* = 0.003) and HI (72.5% vs. 25.0%, *p* < 0.001) had significantly higher survival rate than that of the NC group. Since intervention was not independent of gender and outcome, the confounding effect of intervention need to be considered when investigating the correlation between gender and outcome. Mantel–Haenszel stratified analysis revealed that gender was associated with outcome when intervention was considered as confounders (O.R. 2.611, 95% C.I. [1.16 5.87], *p* = 0.020).

**Table 4:** Three-dimensional contingency table relating 96 h outcome to gender in animals treated with different post resuscitation interventions

Intervention		NC			TTM			HI		
Outcome		Survived	Died		Survived	Died		Survived	Died	
Gender	Male	3	17		11	9		11	9	
	Female	7	13		12	8		18	2	

*NC* Normothermic control, *TTM* targeted temperature management, *HI* hydrogen inhalation

**Table 5:** Mutual independence testing results among gender, intervention and survival

Hypothesis (H0)	Chi-square statistic	Degree of freedom	Critical value	Result
Gender, intervention and outcome are mutual independent	25.5	7	14.1	*p* < 0.05, reject H0
Outcome is independent of gender and intervention	25.5	5	11.1	*p* < 0.05, reject H0
Gender is independent of intervention and outcome	8.4	5	11.1	*p* > 0.05, accept H0
Intervention is independent of gender and outcome	21.7	6	12.6	*p* < 0.05, reject H0

## Discussion

The present study investigated the interaction between gender and post resuscitation intervention on neurological outcome in an adult rat model of cardiac arrest and CPR. Our results demonstrated that gender did not affect resuscitation but associated with neurological outcome. The superiority of female rats in neurological recovery was influenced by post resuscitation interventions. Specifically, females and males exhibited equivalent response to hypothermia. But there was a difference in the response to hydrogen in females and males, and female rats were more likely to benefit from hydrogen therapy.

Despite decades of research and major investment, survival after OHCA remains poor. Innate physiological differences between men and women prompt researchers to investigate the influence of gender on the outcome of CPR. Perers et al. [7] reported that female gender was associated with an increased chance of ROSC, although women differ from men in being older, receiving bystander CPR less frequently, and being found in ventricular fibrillation less frequently. Two subsequent retrospective cohort studies supported Perers’ report that women had higher resuscitation rate than men, but found the difference in resuscitation rate only presented in reproductive ages [15, 16]. However, Lewis et al. [17] observed that women were significantly less likely to be resuscitated compared with men using 284,000 OHCA activations data from the National Emergency Medical Services Information System. A recent meta-analysis involving patient from 15 studies showed that there was no significant difference in hospital admission between females and males. The authors therefore speculated that some of the pre-hospital baseline characteristics such as age, bystander CPR, and shockable rhythm had no obvious immediate impact did not have an impact on the difference in resuscitation rate between the genders [6].

In order to determine the difference in gender related to the survival at hospital discharge, Herlitz et al. [8] analyzed 23,797 patients (27.9% women) in the Swedish Cardiac Arrest Registry. They observed that female gender was associated with an increased survival and was an independent predictor for being hospitalized alive. Safdar et al. [18] observed that the probability of survival differed across age for men and women in a nonlinear fashion: women had a higher survival probability before the age 47, after which men had a higher survival probability. By analyzing a nationwide population-based OHCA database involving 318,123 patients (40.8% females) in Japan, Akahane et al. [19] reported that the rate of survival with favorable neurologic outcome was significantly higher for women than men in the group aged 40 to 59 years. On the contrary, Bosson et al. [9] reported that gender was not associated with survival or neurological outcome after OHCA with adjustment for factors of characteristics and treatments. Mahapatra et al. [20] reported that women had significantly lower survival to discharge than men in patients with VF OHCA. Karlsson et al. [21] reported that women not only had a lower survival rate, but also had poor neurological outcome than that of men. There are two possible explanations for gender-based disparities for survival. On the one hand, there was considerable heterogeneity in patient selection among the aforementioned studies, and the sample size of these who suggested that women had a higher survival was much larger than those who did not [8, 9, 18–21]. On the other hand, women victims were less likely to receive post resuscitation interventions, such as coronary angiography, percutaneous coronary intervention, and TTM [5, 6, 22, 23].

To determine the effect of gender differences on outcome of TTM, Greenberg et al. [24] compared the mortality rate at discharge among patients who received TTM after resuscitation. The authors reported that women were 54% less likely to die than men after adjusting for confounders. In another study, however, Oh et al. [25] reported that the neurological outcomes of women worsened from 18 to 59 years of age in contrast to the outcomes in males. Fillbrandt et al. [26] reported that patients who underwent TTM did not show any gender differences in benefits from hypothermia. Furthermore, Winther-Jensen et al. [27] reported that there was no interaction between gender and the effect of targeting 33 °C or 36 °C for 24 h. Since the underlying disease, comorbidities and treatment characteristics were inconsistent among these studies, more research still need to be invested in gender related aspects of treatment in order to provide the best intensive care to the post resuscitation patients.

This is the first animal study to systematically investigate the interaction between gender and post resuscitation interventions. An asphyxia cardiac arrest model was used because the majority of the OHCA patients are found to be in a non-shockable initial rhythm, and cardiac arrest with non-shockable rhythm was associated with a poorer neurologic prognosis than cardiac arrest with shockable rhythm [7, 16]. TTM was utilized as an intervention because it is currently recommended as a routine treatment measure for all cardiac rhythms in both out-of-hospital and in-hospital cardiac arrest by the latest Guidelines [4]. Hydrogen was utilized as additional intervention because it is demonstrated to be superior to TTM for improving neurological outcome and survival in our previous animal studies [11, 28]. For animals treated with normothermia, although females had significantly lower MAP and longer EEG recovery time, but the survival rate was relatively higher than that of the males. This contradicted with the findings that a higher blood pressure and shorter EEG recovery time associated with better outcome [29, 30]. This inconsistency further supported the presence of clinically-relevant pathophysiological differences between males and females. Indeed, numerous animal studies have validated that the gender difference in neurological outcome was due primarily to the high levels of circulating estrogen in adult female animals: removal of endogenous sex steroids increased female brain injury, whereas exogenous estrogen administered to males and females reduced ischemic neuronal injury [31]. For animals treated with hypothermia, the differences in MAP and EEG between females and males were reduced. The finding that post resuscitation hypothermia protected both males and females with equivalent effectiveness was consistent with Dietz et al. [32] even though a different animal model was used in this study. For animals treated with hydrogen, the differences in MAP and EEG between females and males were also reduced, as that was shown in the TTM group. This suggests that female rats and male rats respond differently to post resuscitation interventions. The differences in NDS and survival rate between females and males further indicated that the beneficial effect of hydrogen was gender dependent. For female rats, an elevated MAP after resuscitation could improve cerebral blood flow through increased cerebral perfusion pressure, potentially attenuating ongoing cerebral injury [29]. The influence of gender on the neurological outcome of TTM and hydrogen may explain by the underlying mechanisms. The neuroprotective effect of hypothermia was through the decrease in metabolic rate and the reduction of cerebral blood flow [33]. On the contrary, the neuroprotective effect of hydrogen was primarily through selective reactive oxygen species attenuation [34]. Although females and males experience the same process of ischemia and reperfusion injury, but perhaps the injury in females is more responsive to hydrogen because the high levels of sex hormones present in adult females may facilitate the treatment [15]. The female specific beneficial effect of hydrogen was also reported in a mouse of Alzheimer’s disease model, and Hou et al. [35] showed that hydrogen-rich water ameliorated oxidative stress and inflammatory responses more profoundly in the brains of female mice than in those of males. The potential mechanism of the gender dependent effect of hydrogen was through estrogen—estrogen receptor β-brain-derived neurotrophic factor signaling. Additionally, female rats of the same age had significantly lower body weight than males. The smaller size led to decreased dosages of pentobarbital and epinephrine, thus might provide benefit for resuscitation and neurological recovery [36, 37]. Therefore, the pathologic or hormonal influence between males and females should be considered when evaluating neurological outcome and combining estrogen may potentiate the effectiveness of post resuscitation interventions.

Some limitations have to be taken into account in our study. First, the animal models did not imitate the clinical scenario of cardiac arrest completely, since the study was performed in healthy animals without underlying cardiac diseases. Second, this was an observational study and the potential mechanism of the gender specific beneficial effect of hydrogen was undetermined. Third, we could not perform histological staining to show the ischemic neuronal changes between male and female rats because the organs and tissues of survived animals were not harvested in this study. Fourth, the proposal that the combination of estrogen and post resuscitation intervention may enhance the efficacy needs to be verified in our future studies.

## Conclusions

In this rat model cardiac arrest and CPR, gender did not affect resuscitation but associated with neurological outcome. The superiority of female rats in neurological recovery was affected by post resuscitation interventions and female rats were more likely to benefit from hydrogen therapy.

## Supplementary Information

**Additional file 1.** Representative raw EEG tracing before and after cardiac arrest (Fig. S1) and neurological deficit score (Table S1).

## Data Availability

The datasets used and/or analyzed during the study are available from the corresponding author on reasonable request.
