# Supplementary material for: Interaction between gender and post resuscitation interventions on neurological outcome in an asphyxial rat model of cardiac arrest
Source: BMC Cardiovasc Disord. 2021 Sep 16;21:441. doi: 10.1186/s12872-021-02262-5 (PMC8443961; doi:10.1186/s12872-021-02262-5)
Supplement: Supplementary file 1 — Additional file 1. Representative raw EEG tracing before and after cardiac arrest (Fig. S1) and neurological deficit score (Table S1). [file 12872_2021_2262_MOESM1_ESM.docx]

**Supplemental figure. 1** Representative raw electroencephalogram (EEG) tracing before and after cardiac arrest. (A) Normal EEG during baseline measurement. (B) isoelectric EEG recorded 10 minutes after resuscitation. (C) Burst suppression EEG was observed 18 min after resuscitation, so the onset time of the EEG burst (OTOB) was 18 min. (D) Burst suppression EEG recorded 100 min after resuscitation. The showed a discontinuous signal because there of the exits of suppression. (E) Continuous EEG pattern was observed 142 min after resuscitation, so the time to normal EEG trace (TTNT) was 142 min.

**Supplemental figure 1**


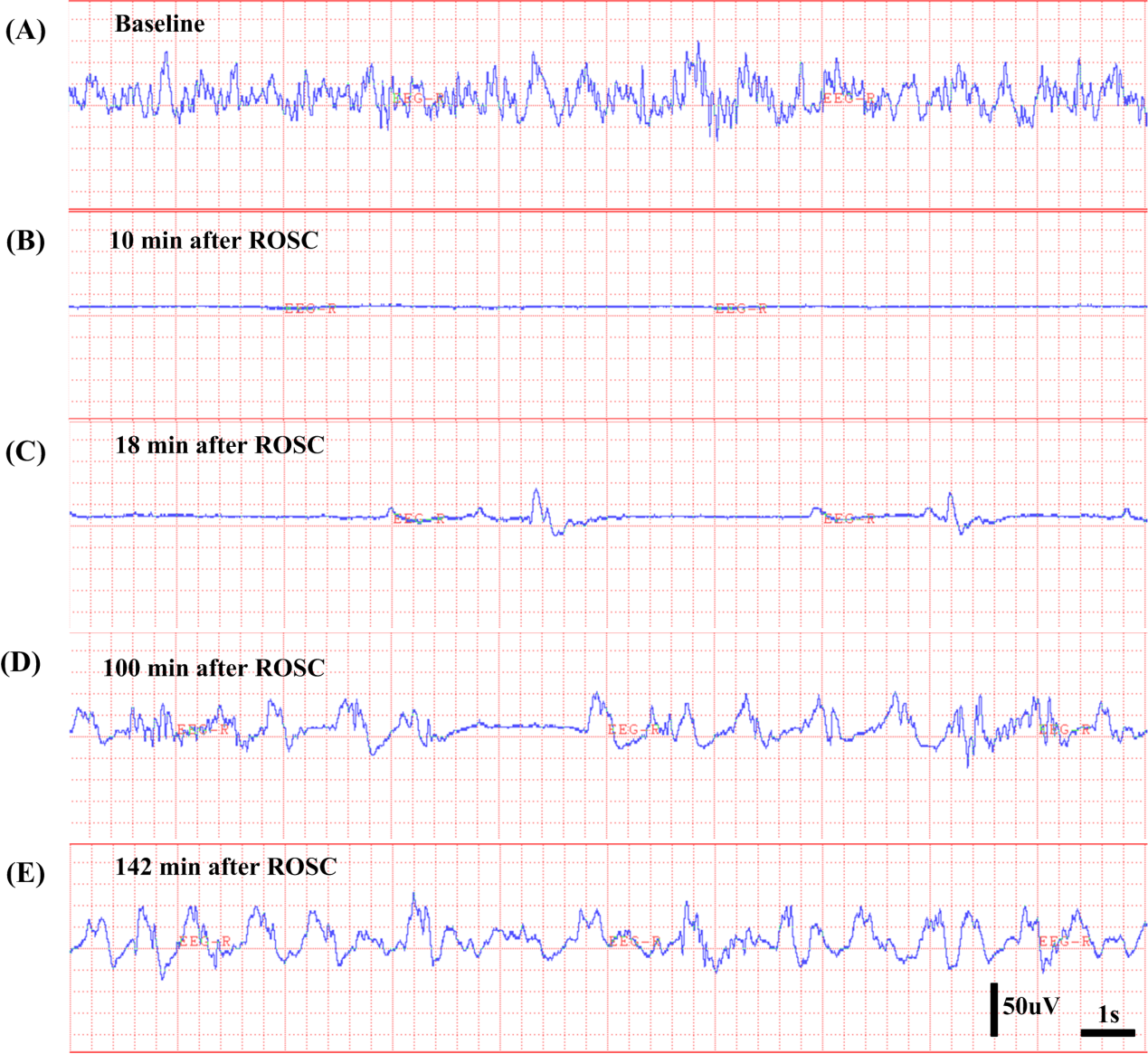


Supplemental Table 1 Neurological deficit score (median and interquartile range)

| Time | Gender | NC (n=40) | TTM (n=40) | HI (n=40) |
| --- | --- | --- | --- | --- |
| At 24 hour | Male (n=20) | 395 (299, 500) | 278 (249, 354) | 223 (158, 266) |
|  | Female (n=20) | 315 (236, 425) | 268 (229, 314) | 178 (119, 205)* |
| At 48 hour | Male (n=20) | 500 (364, 500) | 263 (191, 500) | 185 (96, 500) |
|  | Female (n=20) | 490 (208, 500) | 210 (164, 344) | 105 (78, 165)* |
| At 72 hour | Male (n=20) | 500 (500, 500) | 250 (120, 500) | 118 (34, 500) |
|  | Female (n=20) | 500 (134, 500) | 153 (120, 500) | 45 (29, 111)* |
| At 96 hour | Male (n=20) | 500 (500, 500) | 315 (30, 500) | 58 (10, 500) |
|  | Female (n=20) | 500 (73, 500) | 95 (30, 500) | 8 (0, 91)* |

NC: normothermic control; TTM: targeted temperature management; HI: hydrogen inhalation. *: *p*<0.05 versus males.
